# pH-Triggered Molecular Alignment for Reproducible SERS Detection via an AuNP/Nanocellulose Platform

**DOI:** 10.1038/srep18131

**Published:** 2015-12-11

**Authors:** Haoran Wei, Peter J. Vikesland

**Affiliations:** 1 Department of Civil and Environmental Engineering, Virginia Tech, Blacksburg, Virginia; 2 Virginia Tech Institute of Critical Technology and Applied Science (ICTAS) Sustainable Nanotechnology Center (VTSuN), Blacksburg, Virginia; 3 Center for the Environmental Implications of Nanotechnology (CEINT), Duke University, Durham, North Carolina

## Abstract

The low affinity of neutral and hydrophobic molecules towards noble metal surfaces hinders their detection by surface-enhanced Raman spectroscopy (SERS). Herein, we present a method to enhance gold nanoparticle (AuNP) surface affinity by lowering the suspension pH below the analyte pK_a_. We developed an AuNP/bacterial cellulose (BC) nanocomposite platform and applied it to two common pollutants, carbamazepine (CBZ) and atrazine (ATZ) with pK_a_ values of 2.3 and 1.7, respectively. Simple mixing of the analytes with AuNP/BC at pH < pK_a_ resulted in consistent electrostatic alignment of the CBZ and ATZ molecules across the nanocomposite and highly reproducible SERS spectra. Limits of detection of 3 nM and 11 nM for CBZ and ATZ, respectively, were attained. Tests with additional analytes (melamine, 2,4-dichloroaniline, 4-chloroaniline, 3-bromoaniline, and 3-nitroaniline) further illustrate that the AuNP/BC platform provides reproducible analyte detection and quantification while avoiding the uncontrolled aggregation and flocculation of AuNPs that often hinder low pH detection.

Surface-enhanced Raman spectroscopy (SERS) has been proposed for ultrasensitive chemical analyses ever since the technique exhibited the capacity for single molecule detection^1^,^2^. Compared to other analytical techniques, SERS does not necessarily require laborious sample pretreatment nor expensive instrumentation, and thus is promising for rapid field and point of use detection^3^,^4^,^5^. To date, however, the hoped for utilization of SERS for rapid detection of environmental pollutants has yet to be realized due to the challenges and costs associated with the production of reproducible SERS substrates^6^,^7^,^8^ as well as the intrinsic requirement that the analyte consistently associates with the substrate to generate a strong, reproducible Raman signal^9^,^10^. Because of these factors, many SERS studies continue to utilize model analytes with high surface affinity to test assay performance^5^,^11^,^12^,^13^,^14^,^15^,^16^. Unfortunately, many relevant analytes are moderately hydrophobic and thus exhibit low affinity to gold or silver nanoparticle (AuNP or AgNP) surfaces. To overcome this drawback, molecular “traps” have recently been used to bind target molecules to the AuNP/AgNP surface^9^,^17^,^18^,^19^,^20^,^21^. Modification of the noble metal surface with these “traps” adds complexity to the material synthesis and produces a potentially interfering background signal that makes data analysis more challenging. To mitigate this issue, some investigators manipulate electrostatic forces, either by altering the AuNP/AgNP surface coating or adjusting the solution pH to enhance the affinity of the analyte for the plasmonic nanoparticle surface^22^,^23^. Unfortunately, surface coatings can decrease the SERS signal intensity due to the increased distance between the analyte and the surface^24^, and AuNP/AgNP suspensions generally exhibit uncontrolled aggregation and flocculation at low pH values and are thus inappropriate SERS enhancers under these conditions. For analytes with low pK_a_ values it is necessary to develop a SERS platform that is stable at low pH.

Bacterial cellulose (BC) is a low-cost bacterial by-product that is biodegradable and exhibits minimal to no toxicity^25^. Compared to common cellulosic materials, BC fibers are nanoscale in radial diameter (<100 nm) and are tightly interwoven as a layered high mechanical strength hydrogel^25^,^26^. Importantly, unlike paper, BC retains its 3D structure in water and is resistant to both acidic and alkaline pH^27^. Recently, its role as a scaffold for nanoparticles for SERS and other applications has been realized^27^,^28^,^29^. Our prior studies with this nanocomposite suggest that the coupling of AuNPs with BC may be a promising approach for the development of a stable low pH SERS platform since the AuNPs are immobilized within the BC matrix and both components are acid-resistant.

In this study, AuNP/BC nanocomposites were synthesized by boiling HAuCl_4_-treated BC in sodium citrate (Na_3_Cit) solution to produce AuNPs that are fully intercalated within the BC network. These nanocomposites were then tested as a SERS platform under acidic pH conditions. To illustrate the capacity of this substrate, two common environmental contaminants - carbamazepine (CBZ; pK_a_ = 2.3) and atrazine (ATZ; pK_a_ = 1.7) - were used as model, environmentally relevant analytes^30^,^31^,^32^,^33^. CBZ is an micropollutant of emerging concern and is one of the most frequently detected pharmaceuticals in surface water^32^. ATZ is widely used for weed control in corn acreage and is the most frequently detected herbicide in surface water^31^. Each of these analytes are moderately hydrophobic (K_ow_ > 1.5; refs. 34,35) with low pK_a_ values and exhibit low AuNP surface affinity under neutral pH conditions.

## Results

### Material characterization

**Figure 1:**
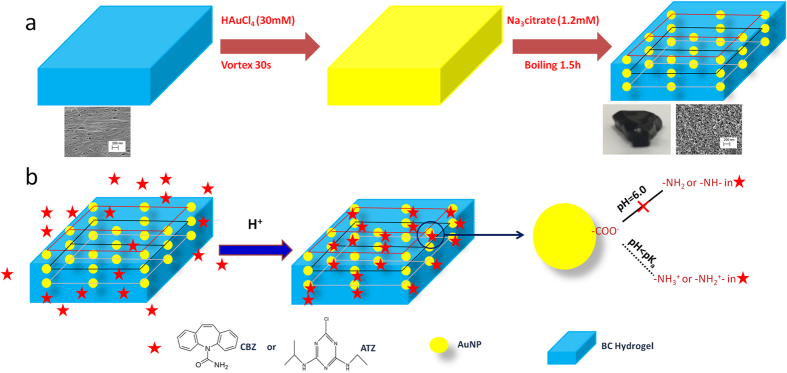
(**a**) Schematic of synthesis of AuNP/BC nanocomposites and (**b**) schematic of pH-induced adsorption of CBZ and ATZ on AuNP/BC.

Our AuNP/BC nanocomposite was synthesized by vortexing BC in 0.7 mL of 30 mM HAuCl_4_ and then boiling in 50 mL of 1.2 mM Na_3_Cit. As shown schematically in Fig. 1a, the as produced AuNP/BC nanocomposite is a rigid hydrogel with large numbers of 63 ± 17 nm (n = 200) diameter AuNPs widely distributed throughout the BC matrix (Supplementary Fig. S-1a&b). The extinction spectrum shows a broad LSPR band with two maxima at 589 and 637 nm indicating the *in situ* formed AuNPs are highly aggregated (Supplementary Fig. S-1c). The AuNP/BC nanocomposite exhibits an extremely flat surface topography that facilitates XY image scan collection (Supplementary Fig. S-1d). Furthermore, the AuNP/BC platform is remarkably stable as shown by a pH invariant extinction spectrum (Supplementary Fig. S-1c) and the lack of any detectable variation in AuNP size (Supplementary Fig. S-2) following exposure to either neutral or acidic pH. This stability is reflected by the consistency of our previously acquired MGITC SERS spectra at neutral and acidic pH^27^. Compared with suspension-based SERS, AuNP/BC provides a rigid scaffold that prevents uncontrolled aggregation and flocculation and thus has potential for use as a low pH SERS substrate.

### pH-triggered SERS

Many analytes used to test novel SERS substrates (e.g., rhodamine 6G, crystal violet, Nile blue, etc.) are positively charged at neutral pH and either covalently or electrostatically associate with negatively charged AuNPs. However, both CBZ and ATZ are neutral molecules at environmental pH and thus exhibit low affinity to the AuNP surface due to the lack of an electrostatic attraction. No Raman signal for either CBZ or ATZ could be observed following exposure of an AuNP suspension to CBZ or ATZ at pH = 6.0 (Supplementary Fig. S-3). CBZ and ATZ contain primary and secondary amine groups, respectively, that are protonated at pH values below their respective pK_a_ values (Fig. 1b). Unfortunately, under these pH conditions many AuNPs uncontrollably aggregate and are thus unsuitable for use as SERS enhancers^22^. We speculated, however, that the stability of AuNP/BC at low pH would enable the protonated amine groups of CBZ and ATZ to associate with the carboxylate groups of AuNP bound citrate via electrostatic attraction (Fig. 1b) and that this would facilitate their SERS detection.

**Figure 2:**
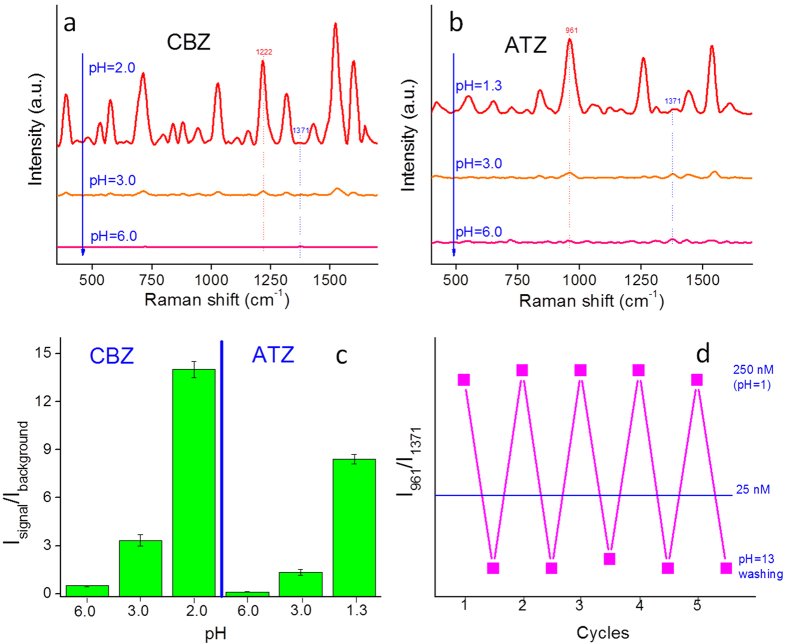
Average Raman spectra of AuNP/BC exposed to (a) 250 μM CBZ and (b) ATZ solutions of different pH; (c) Change of signal/background ratio (I_1222_/I_1371_ for CBZ and I_961_/I_1371_ for ATZ) as a function of solution pH; (d) Change of signal/background ratio for ATZ with five consecutive exposure to ATZ at pH = 1.3 and NaOH washing at pH = 13. (Average of 400 spectra in a 20 μm × 20 μm area, laser 785 nm, 5 mW, 10× objective).

At neutral pH neither CBZ nor ATZ exhibit a detectable SERS signal (Fig. 2a,b, Supplementary Fig. S-4). However, when the pH decreases from 6.0 to 3.0, the signal intensity increases and at pH values below an analyte’s pK_a_ there is a substantial enhancement in the SERS signal for both CBZ and ATZ. To quantify the influence of pH on the SERS signal, the intensity of the CBZ peak at 1222 cm^−1^ and the intensity of the ATZ 961 cm^−1^ peak were used to reflect the signal from the two compounds (*I*_signal_), while the small peak at 1371 cm^−1^ was used to represent the BC support (*I*_background_). As shown in Fig. 2c, the ratio of the peak intensity for the analyte relative to the background increased 47× for CBZ and 68× for ATZ with a decrease in pH from 6.0 to below the analyte’s pK_a_. Because of the chemical and colloidal stability of the AuNPs restrained within the BC scaffold, AuNP aggregation cannot account for this pH-induced Raman signal enhancement (Supplementary Fig. S-1c and Fig. S-2). Furthermore, the stability of the Raman band at 1371 cm^−1^ under varying pH conditions supports our contention that the AuNP/BC platform is stable at acidic pH (Fig. 2a,b). We did not observe any temporal variations in signal intensity, thus indicating the platform did not degrade at acidic pH. We conclude that the significant SERS enhancement for CBZ and ATZ at low pH is due to an increase in their surface affinity. For pH < pK_a_, the protonated -NH_2_ and -NH- groups in CBZ and ATZ facilitate analyte association with the negatively charged carboxylate groups of the AuNP citrate coating (Fig. 1b). To affirm this speculation, benzoic acid (pK_a_ = 4.2), an aromatic compound without an amine group, was used as a negative control. Under similar conditions as used for CBZ, no Raman signal for BA could be observed, thus further suggesting the role of pH sensitive amine groups in the SERS detection of CBZ and ATZ (Supplementary Fig. S-5). In contrast, our positive control MGITC, which associates with AuNP via a thiol linkage, exhibited pH insensitive SERS^27^,^36^.

**Figure 3:**
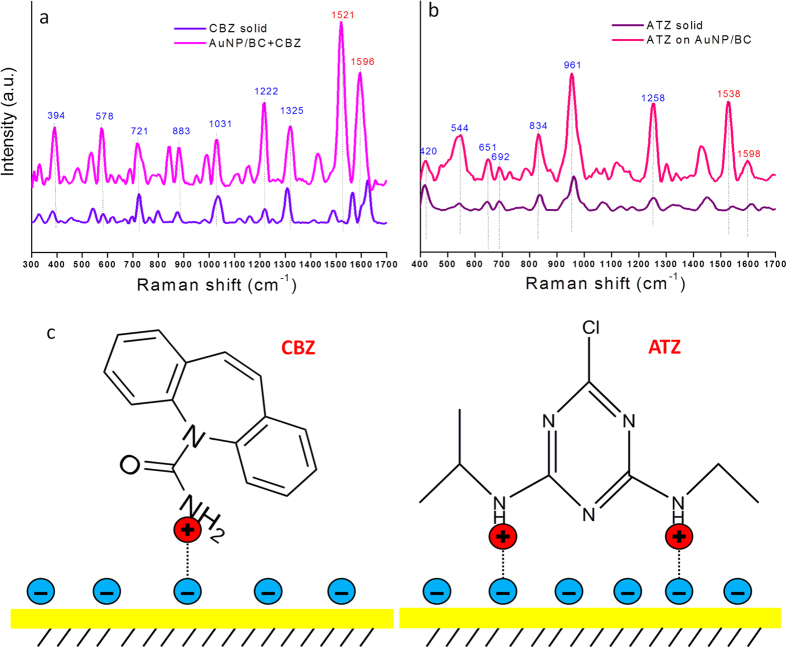
(**a**) Average Raman spectra of CBZ solid, and CBZ on AuNP/BC hydrogel; (**b**) Average Raman spectra of ATZ solid, and ATZ on AuNP/BC hydrogel; (**c**) The molecular orientation of CBZ and ATZ on AuNPs. (Average of 400 spectra in a 20 μm × 20 μm area, laser 785 nm, 5 mW, 10× objective; For ATZ solid, 11.1 mW, 100× objective).

To further support our contention that CBZ and ATZ associate with the surface via their nitrogen groups we examined the collected SERS spectra and compared them to the normal Raman spectrum of each compound (Fig. 3a,b). For each analyte, the prominent peaks in the normal Raman spectra appear in the respective SERS spectra, but exhibit substantial signal enhancements, thus validating that the spectra reflect the SERS of the target analytes. The measured Raman shift reflects the vibrations of chemical bonds, and thus significant differences in Raman shift between the SERS spectrum and the normal Raman spectrum indicate the change in the vibration of a particular chemical bond that is caused by an interaction between the analyte and the NP surface^37^,^38^. In the SERS spectra of CBZ and ATZ there is very little shift in the Raman peaks relative to their normal Raman spectra in the 400-1400 cm^−1^ range (these peaks primarily reflect covalent interactions within the six-member rings and C-C bonds)^39^, thus indicating the main mechanism for the SERS of these bands is via long distance electromagnetic enhancement^40^. However, there are detectable blue shifts and significant enhancement of the peaks in the range 1500–1600 cm^−1^ (N-H and C-N bonds)^39^, indicating shorter distance chemical SERS enhancements in addition to the longer range electromagnetic enhancement^38^. SERS spectral analysis thus supports our speculation that CBZ and ATZ associate with the AuNPs through an electrostatic attraction between amine groups and the citrate coating of the AuNPs. The -NH_2_ group in CBZ and the two protonated -NH- groups of the ATZ ring substituents are in close proximity to the AuNP surface, while the six-member rings are further away from the AuNP surface (Fig. 3c). Our speculation that the positively-charged amine groups of CBZ and ATZ associate with the negatively-charged carboxylate groups on AuNP surface is supported by the following: 1) The significant enhancement in the SERS intensity of CBZ and ATZ was observed at pH values below their respective pK_a_ values, thus highlighting the important role of positively-charged amine groups (Fig. 2). 2) A similar phenomenon was observed for a variety of amine-containing compounds (positive controls) with low pK_a_ values, but not for carboxylate-containing compounds (negative controls; Supplementary Table S-3 and Fig. S-5), further corroborating the role of positively-charged amine groups. 3) The substantial enhancement and shift of the amine SERS bands of CBZ (1521 cm^−1^ and 1596 cm^−1^) and ATZ (1538 cm^−1^ and 1598 cm^−1^) relative to their normal Raman bands indicate that the amine groups were close to the AuNP surface and thus subject to chemical enhancement.

The SERS enhancement factor (EF) for AuNP/BC was estimated using equation (1) and the simplifying assumption that all of the CBZ and ATZ initially added associated with the AuNPs:


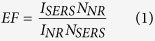


where *I*_SERS_ and *I*_NR_ are the peak intensities at 1222 cm^−1^ for CBZ and 961 cm^−1^ for ATZ on AuNP/BC and solid, respectively, and *N*_SERS_ and *N*_NR_ are estimates for the number of analyte molecules within the laser probe volume for both AuNP/BC and the solid. Using this relationship EF was calculated to be 1 × 10^5^ for CBZ and 3 × 10^5^ for ATZ. We note that our calculated EF is a lower bound of the actual EF due to that fact that substantial amounts of free CBZ and ATZ both remain in solution and associate with the BC matrix. As stated in the highly cited and comprehensive study of SERS EF by Etchegoin and colleagues^41^, an EF of 10^7^ is sufficient to enable single molecule SERS detection and thus our calculated value of 10^5^ reflects a substantial enhancement. We note that a recent study^42^ coupling capillary chromatography with SERS for ATZ detection reported an EF value of only 8 and that we experimentally determined an EF of 10 using a commercially available substrate (Supplementary Figs S-6,7; Note 1). Our value is nearly five orders of orders of magnitude larger. Large variations in the reported SERS spectra for atrazine in the literature and their obvious differences relative to the normal Raman and theoretical spectra of atrazine illustrate the challenges that to date have plagued reproducible atrazine detection by SERS^39^,^43^,^44^,^45^. Furthermore, the lack of information about temporal and spatial SERS signal variations makes the reproducibility of these studies questionable.

### Reproducibility, Reusability, and Quantitation

**Figure 4:**
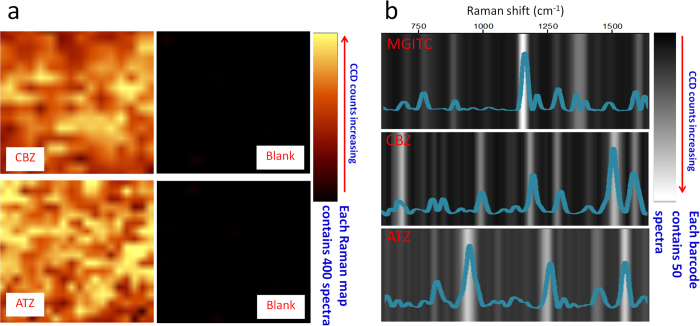
(**a**) Raman XY maps of CBZ, ATZ and blank solutions at pH = 2.0 or 1.3 on AuNP/BC nanocomposite; (**b**) SERS barcodes of 50 randomly selected spectra in a Raman map overlapping together for MGITC, CBZ and ATZ.

The electrostatic attraction between the amine groups of CBZ and ATZ and the AuNP surface results in spatially and temporally repeatable binding of these analytes to the nanocomposite as evinced by both XY Raman imaging as well as SERS barcodes. We produced XY image scans by tracking the intense peaks at 1222 cm^−1^ (CBZ) and 961 cm^−1^ (ATZ). Prior to adding CBZ or ATZ, the Raman maps were completely blank thus demonstrating no observable signal (Fig. 4a). After adding CBZ or ATZ, the maps become bright yellow indicating strong CBZ and ATZ signals (Fig. 4a). The uniform distribution of the SERS signal across the Raman maps illustrates the homogeneity and reproducibility of the binding. To further illustrate the extreme reproducibility of our substrate we constructed “SERS barcodes” for each analyte. Such barcodes have recently been proposed as a tool to succinctly illustrate the relative intensities of all the peaks in a set of collected Raman spectra^46^. Herein we randomly selected 50 spots from our collected XY Raman maps (Fig. 4b). As shown in Fig. 4b, higher intensity Raman bands are brighter, while lower intensity bands are darker. For both CBZ and ATZ perfect barcodes were readily obtained. For comparison, we illustrate the barcode for the MGITC positive control, which was expected to be highly legible because of the covalent Au-S linkage that results in reproducible binding to the surface. For pH < pK_a_, CBZ and ATZ, our two analytes with low surface affinity, exhibited similar reproducible behavior thus further suggesting that they align in consistent orientations across the AuNP surface.

Reusability is important, yet difficult to achieve for many SERS substrates due to the irreversible binding of the analyte to the sensor surface. However, because this platform relies on electrostatic attractions it is possible to regenerate and reuse it by simply cycling the pH between 1.3 and 13 (Fig. 2d). As shown, a consistent SERS signal intensity for ATZ at pH 1.3 is easily recovered through five cycles. This capacity is the result of the acidic and alkaline resistance of the BC matrix as well as the AuNPs.

**Figure 5:**
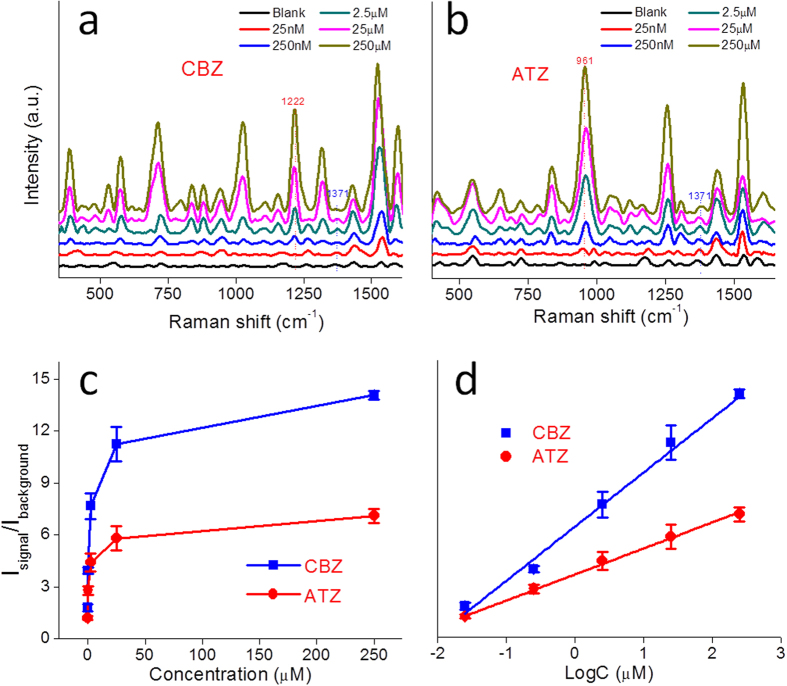
Average Raman spectra for (a) CBZ and (b) ATZ concentrations of 25 nM–250 μM on AuNP/BC hydrogel; (c) The 1222 cm^−1^/1371 cm^−1^ ratio and the 961 cm^−1^/1371 cm^−1^ ratio increase as a function of analyte concentration; (d) The 1222 cm^−1^/1371 cm^−1^ ratio and the 961 cm^−1^/1371 cm^−1^ ratio increase linearly with analyte concentration in logarithmic form.

Using the intensity of the background band at 1371 cm^−1^ as an internal standard we established the minimum quantification level for CBZ and ATZ using the aforementioned peaks at 1222 cm^−1^ and 961 cm^−1^. Accordingly, intensity ratios for signal and background peaks (I_1222_/I_1371_ and I_961_/I_1371_) were used for CBZ and ATZ quantification, respectively. For each analyte a series of concentrations from 25 nM to 250 μM were used to test the AuNP/BC substrate. Over this concentration range the background peak at 1371 cm^−1^ exhibits little change in intensity, while the CBZ and ATZ peaks at 1222 cm^−1^ and 961 cm^−1^ increase monotonically (Fig. 5a,b). For both analytes, the intensity ratio increased significantly from 25 nM–25 μM and then began to level off between 25–250 μM (Fig. 5c). We attribute this latter phenomenon to nanocomposite saturation at high CBZ and ATZ concentrations. When the concentration was plotted in logarithmic form, a linear relationship results (Fig. 5d). The limits of detection (LOD; defined as the signal-to-noise ratio = 3; ref. 47) are 3 nM and 11 nM for CBZ and ATZ, respectively. US EPA has set a regulation limitation for ATZ in drinking water of 3 μg/L. Although our LOD for ATZ (11 nM or 2.3 μg/L) by SERS is slightly higher than that of GC-MS (0.12 μg/L, EPA standard method)^48^, the SERS method is considerably more rapid, is easier and shows potential for on-site detection.

To illustrate the possibility to employ AuNP/BC for real environmental samples, it was tested using surface water acquired from a local creek. For this effort, 100 nM of CBZ and 250 nM of ATZ were spiked into unpurified surface water. Following 15 s of mixing of AuNP/BC with the solution, the nanocomposite was taken out for Raman testing. From the sample spiked with low concentration CBZ (100 nM) and ATZ (250 nM), the characteristic bands of CBZ (394, 578, 1030, 1222, 1325 cm^−1^) and ATZ (544, 651, 692, 834, 961, 1258, 1538 cm^−1^) were clearly observed while from the blank sample no such bands appeared (Supplementary Fig. S-8). These results indicate that AuNP/BC can be applied in real world surface waters.

### Hydrogel deformation-induced intensity changes

**Figure 6:**
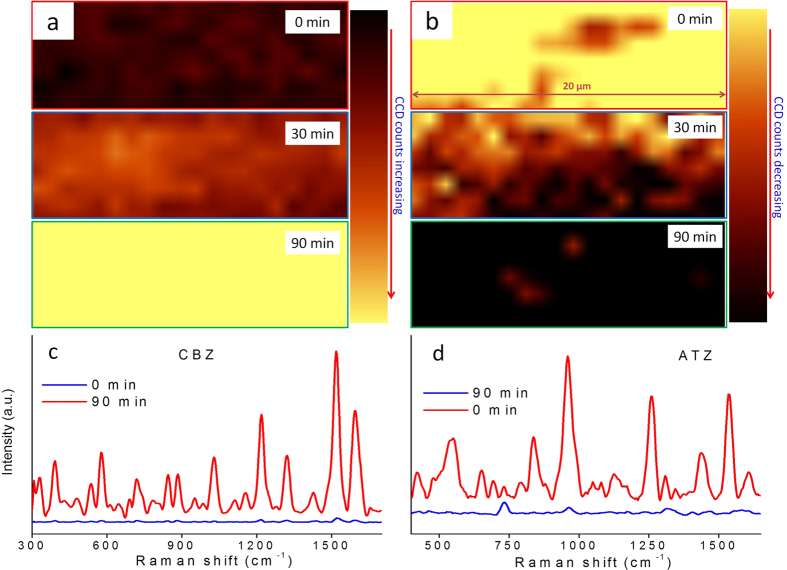
Raman XY maps of the (a) CBZ 1222 cm^−1^ peak and (b) ATZ 961 cm^−1^ peak for the AuNP/BC hydrogel under wet (0 min and 30 min) and dry (90 min) conditions; (Average of 140 spectra in a 20 μm × 7 μm area, laser 785 nm, 5 mW, 10× objective.) Average Raman spectra of (c) CBZ and (d) ATZ for the AuNP/BC hydrogel under wet and dry conditions.

In the hydrated state, AuNP/BC is a three-dimensional hydrogel with a height 5500 ± 100 μm. When fully dried the hydrogel shrinks in the vertical direction into a thin film with a height of 16 ± 1 μm. In our previous study, the drying of AuNP/BC produced significant signal enhancements for MGITC and Rhodamine 6G due to drying induced formation of SERS “hot spots”^27^. Interestingly, CBZ exhibited similar behavior, while ATZ did not. As shown in Fig. 6a, the SERS intensity of CBZ gradually increased from 0 (wet) to 90 min (dry) indicating that positively charged CBZ associates with the AuNP surface during the drying process. However, for ATZ the Raman signal decreases and ultimately disappears when the drying time is increased from 0 (wet) to 90 min (dry; Fig. 6b).

Spatially averaged spectra for CBZ and ATZ under both wet and dry hydrogel conditions are shown in Fig. 6c,d. The spectra of CBZ at 0 and 90 min are similar except for the observed change in SERS intensity (Fig. 6c), while the ATZ spectrum at 0 min is completely lost at 90 min (Fig. 6d). These data suggest that ATZ was transported away from the AuNP surface during drying due to capillary forces generated by water evaporation. We therefore searched for the ATZ signal near the edges of the dry substrate and found it to be heterogeneously distributed across the edge (Supplementary Fig. S-9a). Collected Raman maps of the edge were highly heterogeneous and could not be used to generate clear SERS barcodes (Supplementary Fig. S-9b).

Clearly, AuNP/BC needs to be hydrated for ATZ detection. The much weaker interaction force between ATZ and the AuNP surface relative to CBZ can be attributed to the following: 1) The secondary amine group of ATZ shows less affinity to the surface carboxylate groups than the primary amine of CBZ; 2) In addition to the amine groups of ATZ, there are also one isopropyl group and one ethyl group. These hydrophobic groups are expected to decrease the affinity of ATZ to the polar surface of AuNPs and may provide steric hindrance that restricts the binding of the secondary amine to the surface carboxylate groups (Fig. 3). In contrast, the primary amine group of CBZ is at the far end of the molecular structure without any alkyl groups around, and thus it has greater capacity to bind to the AuNP surface. 3) The K_ow_ of ATZ (2.68) is higher than that of CBZ (1.51) indicating that ATZ is more than one order of magnitude more hydrophobic than CBZ^34^,^35^. The AuNP/BC platform is extremely hydrophilic due to the large number of hydroxyl groups on the nanocellulose fibers. Due to the capillary forces generated during water evaporation, the more hydrophobic ATZ is more likely to recrystallize from the system (Supplementary Fig. S-7 and Note 1) while the more hydrophilic CBZ is more likely to be retained. The above three factors may act synergistically to result in this phenomenon. In the future, we intend to systematically examine the influence of molecular structure on SERS using a series of chemically similar compounds.

### Broad applicability

To examine the broad applicability of our SERS platform, five additional analytes (melamine, 2,4-dichloroaniline, 4-chloroaniline, 3-bromoaniline, and 3-nitroaniline) with low pK_a_ values were tested under both neutral and the pH < pK_a_ conditions (Supplementary Table S-3). For these compounds, the signal/background ratio (I_signal_/I_background_) increased by 2–136× at pH < pK_a_ compared to neutral pH (Supplementary Table S-3), further supporting the pH-triggered affinity enhancement of compounds with amino groups. SERS barcodes (Supplementary Fig. S-10) of these compounds were acquired under low pH conditions thus demonstrating the perfect reproducibility of their SERS spectra. These results further indicate that pH-triggered SERS using the AuNP/BC platform can be applied to a range of pollutants.

## Discussion

A facile and stable AuNP/BC nanocomposite was synthesized and used as a low pH SERS substrate. SERS detection of carbamazepine and atrazine was achieved using this platform by lowering the solution pH to a value below the analyte’s pK_a_. The enhanced affinity and higher SERS intensity are triggered simply by adjusting solution pH without the need to modify the AuNP surface. The electrostatic interaction between the analyte and the AuNP surface can be reversed by adjusting the solution pH. At pH < pK_a,_ the consistent molecular alignment on the AuNP surface results in highly reproducible SERS spectra. This protocol simplifies and reduces the cost of detecting amine-containing compounds with low pK_a_. The sampling and detection time is short (<1 min) and the preparation procedure is simple, thus making this platform promising for real world application. Compared with conventional suspension-based SERS substrates, AuNPs are restrained in BC matrix and are not subject to uncontrolled aggregation and flocculation at acidic pH. Meanwhile, the AuNP/BC is readily stored and transported due to its small volume. Compared with solid SERS substrates, AuNP/BC is easier and cheaper to synthesize and shows much higher EF values and improved reproducibility (Supplementary Fig. S-6&7 and Note 1). Because AuNP/BC is a 3D hydrogel, drying-induced deformation generates additional SERS hot spots in the vertical direction and can lead to stronger SERS intensities for relatively hydrophilic compounds. After drying, the AuNP/BC SERS platform shrinks into a thin (16 μm) and light (0.4 mg) film, which reduces waste compared with solid SERS substrates. This is the first report on the pH-triggered SERS detection of real contaminants with relatively low affinity to AuNP surface. Quantitative analysis was achieved using common citrate-AuNPs without any surface modification. Different from the mainstream literature focusing on the design of intricate nanostructures to enhance the intrinsic SERS EF, this paper emphasizes the importance of surface affinity, especially electrostatic interactions for SERS detection of real-world contaminants. We expect that the AuNP/BC nanocomposite will serve as an ideal platform for studying the influence of solution pH on SERS detection of a broad suite of analytes.

Recently reported SERS substrates with AuNPs embedded in polymeric matricies are summarized in Table S-4^27^,^49^,^50^,^51^,^52^,^53^,^54^,^55^,^56^,^57^. We focus on flexible SERS substrates due to their intrinsic advantages for real world application relative to rigid substrates^58^. Our AuNP/BC platform is compared to existing AuNP/polymer composites in terms of the following: 1) Support material: Compared with cellulose and synthetic polymer nanofibers, the nanocellulose used in this study is a naturally produced polymer that not only can house greater numbers of AuNPs due to its nanoscale size, but also exhibits the potential for biocompatible application. 2) Preparation method: Most AuNP/polymer composites are synthesized by multi-step methods. First, the AuNPs are synthesized based on an existing protocol. Second, the polymers are impregnated by exposure to an AuNP suspension or the polymer precursors are polymerized onto the AuNPs by gelation or electrospinning. Our AuNP/BC nanocomposite was synthesized by a one step *in situ* reduction method that is considerably faster and easier. 3) Application form: A majority of the reported AuNP/polymers have only been used under dry conditions due to their low stability in water. The AuNP/BC platform is a rigid hydrogel with high water stability. It can accumulate analytes directly from water and be reused many times without losing its stability. The hydrogel can “replicate” the pH of the bulk solution that is the basis for our later pH-triggered detection. 4) Application pH: Due to the chemical and water stability of AuNPs (compared with AgNPs) and nanocellulose, our material can be used at acidic pH (as low as 1.3 for ATZ detection) while the other materials in Table S-4 have only been tested at neutral pH. At low pH values, amine-containing compounds with low pK_a_ are protonated and easily associate with the citrate-coated AuNP surface. This low-pH stable SERS platform extends the application of SERS to the detection of low pK_a_ compounds in an easy and reproducible manner. Further, by manipulating solution pH, this material can be easily regenerated and reused, which to our knowledge cannot be achieved by other AuNP/polymer materials. 5) Target analyte: As indicated in Table S-4, most of the AuNP/polymer substrates reported to date have been tested using Raman resonant dyes or compounds with sulfur atoms. These compounds either exhibit large Raman cross sections or have a strong affinity to the AuNP surface. In our study, two neutral environmental contaminants - atrazine and carbamazepine with low affinity to AuNPs were tested. Excellent SERS reproducibility and sensitivity were achieved for these compounds by simply adjusting solution pH.

## Methods

### Reagents

Gold chloride trihydrate (HAuCl_4_ · 3H_2_O) was purchased from MP Biomedicals. Sodium citrate tribasic dihydrate (Na_3_Cit · 2H_2_O), benzoic acid (BA), melamine, 2,4-dichloroaniline, 4-chloroaniline, 3-bromoaniline, and 3-nitroaniline were purchased from Sigma-Aldrich. Atrazine (98.9%) and carbamazepine (99.0%) were purchased from Chem. Service and Acros Organics, respectively. Malachite green isothiocyanate (MGITC) was acquired from Invitrogen Corp. (Grand Island, NY). HCl and ethanol were purchased from Fisher Scientific. BC was grown by culturing *Gluconacetobacter xylinus* in corn steep liquor for 14 days^59^. Surface water was obtained from Tom’s Creek near the Virginia Tech campus and was used without pretreatment.

### **Preparation** of AuNP/BC

Sixteen pieces of BC (0.5 cm × 0.5 cm) were incubated in 0.7 mL HAuCl_4_ solution (30 mM) and vortexed for 30 s. Subsequently, they were transferred into 50 mL of boiling 1.2 mM Na_3_Cit and kept for 1.5 h. The resultant AuNP/BC was rinsed 10× with 25 mL aliquots of DI water. In parallel, a suspension of AuNPs with a uniform size of 50 nm was synthesized via seed-mediated growth (Supplementary Note 2)^60^.

### Control experiments

Control experiments were conducted to exclude potential background interferences in the normal Raman spectra of CBZ and ATZ. CBZ or ATZ (250 μM) when dissolved in ethanol only exhibit the Raman peaks of the ethanol solvent (Fig. S-3) thus indicating the normal Raman spectrum of CBZ and ATZ in solution is challenging to obtain. Under our operating conditions the normal Raman spectrum of CBZ and ATZ could only be acquired using CBZ or ATZ solids and a high energy laser. Assignments of the Raman bands for CBZ and ATZ are found in Supplementary Tables S-1 and S-2. Raman spectra of CBZ and ATZ on pure BC were acquired to verify all the Raman signals obtained previously were from AuNP-enabled SERS. As shown in Supplementary Fig. S-4a,b, no Raman signal was obtained using only BC, thus indicating the spectra originate from AuNP-enabled SERS. Furthermore, exposure of AuNP/BC to solutions without CBZ or ATZ produces only a weak Raman signal that corresponds to citrate (Supplementary Fig. S-4a,b).

### Sampling

To study the influence of pH on SERS, one piece of AuNP/BC was immersed in 4 mL of 250 μM CBZ or ATZ solution at pH = 1.3–6.0 and vortexed for 15 s. As control experiments, AuNP/BC was exposed to 4 mL of 2.5 μM MGITC solution at pH = 6.0 and 250 μM BA solution at pH = 2.0. To quantify CBZ and ATZ, AuNP/BC was immersed in 4 mL 0.025–250 μM CBZ and ATZ solution at pH = 2.0 or 1.3. To test the substrate in real environmental waters, AuNP/BC was immersed in 4 mL surface water spiked with 100 nM CBZ or 250 nM ATZ at pH = 2.0 or 1.3, respectively. A blank surface water sample pH adjusted with HCl was used as a control.

### Regeneration

Following exposure to 4 mL of 250 nM ATZ solution at pH = 1.3, one piece of AuNP/BC was characterized via Raman spectroscopy. Subsequently, the sample was washed 3× with 10 mL DI water, 3× with 2 mL NaOH solution (pH = 13), and copiously washed with DI water to remove NaOH. Following alkaline washing, the sample was immersed in 4 mL HCl solution (pH = 1.3) and tested again with Raman spectroscopy. The whole process described above was repeated five times.

### Comparison with suspension and solid-based substrate

AuNPs with uniform particle size (50 nm) were prepared and used to represent suspension-based SERS. Following addition of 1 mL of 1 mM CBZ or ATZ solution into 3 mL AuNP suspension and vortexing for 15 s, the suspensions were subjected to Raman measurement. A commercial SERS substrate (Klarite) was also tested. A Raman map was obtained at the edge of the coffee ring formed by drop deposition of 10 μL of 1 mM ATZ solution on the Klarite substrate.

### Instrumentation

Following sampling, the AuNP/BC was put onto aluminum foil and tested via Raman spectroscopy (WITec alpha 500R). For each measurement, a XY area (20 μm × 20 μm) of 400 points was scanned. A 785 nm wavelength laser and a 10× objective (5 mW, 0.5 s integration time) were used to collect each spectrum in the Raman map. The signal was transmitted through a 300 gr/mm grating and detected using a Peltier CCD. Unlike the single spectra reported in most of the SERS literature, each of the spectra reported herein represent the average of the 400 baseline corrected (Origin 8.0) spectra in the collected Raman maps. Each of the calculated “SERS barcodes”^46^ contains 50 randomly selected spectra from one XY Raman map. Each spectrum (352 data points) was normalized to the most intense peak before all the data points (352 × 50) were converted into a matrix that was subsequently projected into “barcode” images using Origin 8.0. To monitor Raman signal changes due to the deformation of the AuNP/BC hydrogel, Raman maps were scanned every 30 min. The morphologies of AuNP/BC were characterized by field emission scanning electron microscopy (FESEM, LEO (ZEISS) 1550). Extinction spectra of AuNP/BC were measured with a UV-Vis spectrophotometer (Cary 5000, Agilent) after pasting the hydrogel on the inner wall of a cuvette.

## Additional Information

**How to cite this article**: Wei, H. and Vikesland, P. J. pH-Triggered Molecular Alignment for Reproducible SERS Detection via an AuNP/Nanocellulose Platform. *Sci. Rep.* **5**, 18131; doi: 10.1038/srep18131 (2015).

## Supplementary Material

Supplementary Information

## References

[b1] Nie S. & Emory S. R. Probing single molecules and single nanoparticles by surface-enhanced Raman scattering. Science 275, 1102–1106 (1997). 9027306 10.1126/science.275.5303.1102

[b2] Kneipp K. *et al.* Single molecule detection using surface-enhanced Raman scattering (SERS). Phys. Rev. Lett. 78, 1667 (1997).

[b3] Haynes C. L. *et al.* Surface-enhanced Raman spectroscopy. Anal. Chem. 77, 338 A-346 A (2005).

[b4] Sharma B. *et al.* SERS: materials, applications, and the future. Mater. Today 15, 16–25 (2012).

[b5] Wei H. *et al.* Plasmonic colorimetric and SERS sensors for environmental analysis. Environ. Sci. Nano 2, 120–135 (2015).

[b6] Kahl M. *et al.* Periodically structured metallic substrates for SERS. Sensor. Actuat, B - Chem 51, 285–291 (1998).

[b7] Ho C. C. *et al.* Quasi-3D gold nanoring cavity arrays with high-density hot-spots for SERS applications via nanosphere lithography. Nanoscale 6, 8606–8611 (2014). 24978350 10.1039/c4nr00902a

[b8] Gong J. *et al.* Micro-and nanopatterning of inorganic and polymeric substrates by indentation lithography. Nano Lett. 10, 2702–2708 (2010). 20557080 10.1021/nl101675s

[b9] Dasary S. S. *et al.* Gold nanoparticle based label-free SERS probe for ultrasensitive and selective detection of trinitrotoluene. J. Am. Chem. Soc. 131, 13806–13812 (2009). 19736926 10.1021/ja905134d

[b10] Alvarez-Puebla R. A. & Liz-Marzan L. M. Traps and cages for universal SERS detection. Chem. Soc. Rev. 41, 43–51 (2012). 21818469 10.1039/c1cs15155j

[b11] Cho W. J. *et al.* Ultrahigh-density array of silver nanoclusters for SERS substrate with high sensitivity and excellent reproducibility. ACS Nano 6, 249–255 (2011). 22117916 10.1021/nn2035236

[b12] Rodríguez-Lorenzo L. *et al.* Zeptomol detection through controlled ultrasensitive surface-enhanced Raman scattering.J. Am. Chem. Soc. 131, 4616–4618 (2009). 19292448 10.1021/ja809418t

[b13] Wang H. *et al.* Nanosphere arrays with controlled sub-10-nm gaps as surface-enhanced Raman spectroscopy substrates. J. Am. Chem. Soc. 127, 14992–14993 (2005). 16248615 10.1021/ja055633y

[b14] Alvarez-Puebla R. *et al.* Nanoimprinted SERS-active substrates with tunable surface plasmon resonances. J. Phys. Chem. C 111, 6720–6723 (2007).

[b15] Tan R. *et al.* 3D arrays of SERS substrate for ultrasensitive molecular detection. Sensor. Actuat. A - Phys. 139, 36–41 (2007).

[b16] Tao A. *et al.* Langmuir-Blodgett silver nanowire monolayers for molecular sensing using surface-enhanced Raman spectroscopy. Nano Lett. 3, 1229–1233 (2003).

[b17] Nergiz S. Z. *et al.* Biomimetic SERS substrate: peptide recognition elements for highly selective chemical detection in chemically complex media. J. Mater. Chem. A 1, 6543–6549 (2013).

[b18] Guerrini L. *et al.* Functionalization of Ag nanoparticles with dithiocarbamate calix [4] arene as an effective supramolecular host for the surface-enhanced Raman scattering detection of polycyclic aromatic hydrocarbons. Langmuir 22, 10924–10926 (2006). 17154566 10.1021/la062266a

[b19] Guerrini L. *et al.* Sensing polycyclic aromatic hydrocarbons with dithiocarbamate-functionalized Ag nanoparticles by surface-enhanced Raman scattering. Anal. Chem. 81, 953–960 (2009). 19127991 10.1021/ac801709e

[b20] Álvarez‐Puebla R. A. *et al.* Au@ pNIPAM colloids as molecular traps for surface‐enhanced, spectroscopic, ultra‐sensitive analysis. Angew. Chem. Int. Ed. 48, 138–143 (2009). 10.1002/anie.200804059 19039813

[b21] Guerrini L. *et al.* Nanosensors based on viologen functionalized silver nanoparticles: few molecules surface-enhanced raman spectroscopy detection of polycyclic aromatic hydrocarbons in interparticle hot spots. Anal. Chem. 81, 1418–1425 (2009). 19215145 10.1021/ac8021746

[b22] Alvarez-Puebla R. A. & Aroca R. F. Synthesis of silver nanoparticles with controllable surface charge and their application to surface-enhanced Raman scattering. Anal. Chem. 81, 2280–2285 (2009). 19222226 10.1021/ac8024416

[b23] Alvarez-Puebla R. A. *et al.* Role of nanoparticle surface charge in surface-enhanced Raman scattering. J. Phys. Chem. B 109, 3787–3792 (2005). 16851426 10.1021/jp045015o

[b24] Ye Q. *et al.* Surface-enhanced Raman scattering from functionalized self-assembled monolayers. 2. Distance dependence of enhanced Raman scattering from an azobenzene terminal group. J. Phys. Chem. B 101, 8221–8224 (1997).

[b25] Iguchi M. *et al.* Bacterial cellulose—a masterpiece of nature's arts. J. Mater. Sci. 35, 261–270 (2000).

[b26] Park M. *et al.* Spatial deformation of nanocellulose hydrogel enhances SERS. Biochip J. 7, 234–241 (2013).

[b27] Wei H. *et al.* Preparation and evaluation of nanocellulose–gold nanoparticle nanocomposites for SERS applications. Analyst 140, 5640–5649 (2015). 26133311 10.1039/c5an00606f

[b28] Carpenter A. W. *et al.* Cellulose Nanomaterials in Water Treatment Technologies. Environ. Sci. Technol. (2015). 10.1021/es506351r PMC4544834 25837659

[b29] Wei H. *et al.* Environmental science and engineering applications of nanocellulose-based nanocomposites. Environ. Sci. Nano 1, 302–316 (2014).

[b30] Wang P. & Keller A. A. Sorption and desorption of atrazine and diuron onto water dispersible soil primary size fractions. Water Res. 43, 1448–1456 (2009). 19147172 10.1016/j.watres.2008.12.031

[b31] Hildebrandt A. *et al.* Impact of pesticides used in agriculture and vineyards to surface and groundwater quality (North Spain). Wate r Res. 42, 3315–3326 (2008). 10.1016/j.watres.2008.04.009 18502469

[b32] Tixier C. *et al.* Occurrence and fate of carbamazepine, clofibric acid, diclofenac, ibuprofen, ketoprofen, and naproxen in surface waters. Environ. Sci. Technol. 37, 1061–1068 (2003). 12680655 10.1021/es025834r

[b33] Wei H. *et al.* Regenerable granular carbon nanotubes/alumina hybrid adsorbents for diclofenac sodium and carbamazepine removal from aqueous solution. Water Res. 47, 4139–4147 (2013). 23579087 10.1016/j.watres.2012.11.062

[b34] Solomon K. R. *et al.* Ecological risk assessment of atrazine in North American surface waters. Environ. Toxicol. Chem. 15, 31–76 (1996). 10.1002/etc.2050 23147529

[b35] Scheytt T. *et al.* 1-Octanol/water partition coefficients of 5 pharmaceuticals from human medical care: carbamazepine, clofibric acid, diclofenac, ibuprofen, and propyphenazone. Water, Air, Soil Pollut. 165, 3–11 (2005).

[b36] Qian X. *et al.* Anchoring molecular chromophores to colloidal gold nanocrystals: surface-enhanced Raman evidence for strong electronic coupling and irreversible structural locking. J. Am. Chem. Soc. 134, 2000–2003 (2012). 22257217 10.1021/ja210992b PMC3412403

[b37] Leng W. & Vikesland P. J. MGITC Facilitated Formation of AuNP Multimers. Langmuir 30, 8342–8349 (2014). 24979046 10.1021/la501807n

[b38] Osawa M. *et al.* Charge transfer resonance Raman process in surface-enhanced Raman scattering from p-aminothiophenol adsorbed on silver: Herzberg-Teller contribution. J. Phys. Chem. 98, 12702–12707 (1994).

[b39] Costa J. C. *et al.* Understanding the effect of adsorption geometry over substrate selectivity in the surface-enhanced Raman scattering spectra of simazine and atrazine. J. Phys. Chem. C 115, 4184–4190 (2011).

[b40] Otto A. *et al.* Surface-enhanced Raman scattering. J. Phys.: Condens. Matter 4, 1143 (1992).

[b41] Le Ru E. *et al.* Surface enhanced Raman scattering enhancement factors: a comprehensive study. J. Phys. Chem. C 111, 13794–13803 (2007).

[b42] Carrillo-Carrión C. *et al.* Determination of pesticides by capillary chromatography and SERS detection using a novel Silver-Quantum dots “sponge” nanocomposite. J. Chromatogr., A 1225, 55–61 (2012). 22261222 10.1016/j.chroma.2011.12.002

[b43] Song X. *et al.* Detection of herbicides in drinking water by surface-enhanced Raman spectroscopy coupled with gold nanostructures. J. Food. Meas. Charact. 7, 107–113 (2013).

[b44] Rubira R. J. *et al.* Detection of trace levels of atrazine using surface-enhanced Raman scattering and information visualization. Colloid Polym. Sci. 292, 2811–2820 (2014).

[b45] Bonora S. *et al.* Raman and SERS study on atrazine, prometryn and simetryn triazine herbicides. J. Mol. Struct. 1040, 139–148 (2013).

[b46] Xu J. *et al.* *In situ* strain-level detection and identification of Vibrio parahaemolyticus using surface-enhanced Raman spectroscopy. Anal. Chem. 85, 2630–2637 (2013). 23356387 10.1021/ac3021888

[b47] Rezaee M. *et al.* Determination of organic compounds in water using dispersive liquid–liquid microextraction. J. Chromatogr., A 1116, 1–9 (2006). 16574135 10.1016/j.chroma.2006.03.007

[b48] U.S. Federal Government, Environmental Protection Agency (EPA), EPA Method 523. EPA Document No. 815-R-11-002 http://water.epa.gov/scitech/drinkingwater/labcert/upload/epa815r11002.pdf.Accessed: August 1, 2015.

[b49] Severyukhina A. N. *et al.* Nanoplasmonic chitosan nanofibers as effective SERS substrate for detection of small molecules. ACS Appl. Mater. Interfaces 7, 15466–15473 (2015). 26126080 10.1021/acsami.5b03696

[b50] Zhu H. *et al.* Self-assembly of various Au nanocrystals on functionalized water-stable PVA/PEI nanofibers: a highly efficient surface-enhanced Raman scattering substrates with high density of “hot” spots. Biosens. Bioelectron. 54, 91–101 (2014). 24252765 10.1016/j.bios.2013.10.047

[b51] Zhang C. L. *et al.* Controlled asemblies of gold nanorods in PVA nanofiber matrix as flexible free‐standing SERS substrates by electrospinning. Small 8, 648–653 (2012). 10.1002/smll.201102230 22162434

[b52] Martín A. *et al.* Flexible SERS active substrates from ordered vertical Au nanorod arrays. RSC Adv. 4, 20038–20043 (2014).

[b53] Yao S. *et al.* A highly porous PVA dried gel with gold nanoparticles embedded in the network as a stable and ultrasensitive SERS substrate. Chem. Commun. (Cambridge, U. K.) 49, 6409–6411 (2013). 10.1039/c3cc42726a 23753017

[b54] Shin K. *et al.* Au nanoparticle-encapsulated hydrogel substrates for robust and reproducible SERS measurement. Analyst 138, 932–938 (2013). 23232290 10.1039/c2an35862j

[b55] Lee C. H. *et al.* Highly sensitive surface enhanced Raman scattering substrates based on filter paper loaded with plasmonic nanostructures. Anal. Chem. 83, 8953–8958 (2011). 22017379 10.1021/ac2016882

[b56] Lee C. H. *et al.* Paper-based SERS swab for rapid trace detection on real-world surfaces. ACS Appl. Mater. Interfaces 2, 3429–3435 (2010). 21128660 10.1021/am1009875

[b57] Ngo Y. H. *et al.* Gold nanoparticle–paper as a three-dimensional surface enhanced raman scattering substrate. Langmuir 28, 8782–8790 (2012). 22594710 10.1021/la3012734

[b58] Polavarapu L. & Liz-Marzán L. M. Towards low-cost flexible substrates for nanoplasmonic sensing. Phys. Chem. Chem. Phys. 15, 5288–5300 (2013). 23303134 10.1039/c2cp43642f

[b59] Matsuoka M. *et al.* A synthetic medium for bacterial cellulose production by Acetobacter xylinum subsp. sucrofermentans. Biosci. Biotechnol. Biochem. 60, 575–579 (1996).

[b60] Freus G. Controlled nucleation for the regulation of the particle size in monodisperse gold solutions. Nature (Phys Sci) 241, 20–22 (1973).

