# Supplementary material for: pH-Triggered Molecular Alignment for Reproducible SERS Detection via an AuNP/Nanocellulose Platform
Source: Sci Rep. 2015 Dec 11;5:18131. doi: 10.1038/srep18131 (PMC4676058; doi:10.1038/srep18131)
Supplement: Supplementary Information [file srep18131-s1.pdf]

Supporting Information for

"pH-Triggered Molecule Alignment for Reproducible SESR Detection of Carbamazepine and Atrazine Using a AuNP/Nanocellulose Platform"

Haoran Wei,[1,2,3] and Peter J. Vikesland[1,2,3*]

[1]Department of Civil and Environmental Engineering, Virginia Tech, Blacksburg, Virginia

[2]Virginia Tech Institute of Critical Technology and Applied Science (ICTAS) Sustainable Nanotechnology Center (VTSuN), Blacksburg, Virginia

[3]Center for the Environmental Implications of Nanotechnology (CEINT), Duke University, Durham, North Carolina

*Corresponding author. Phone: (540) 231-3568, Email: pvikes@vt.edu

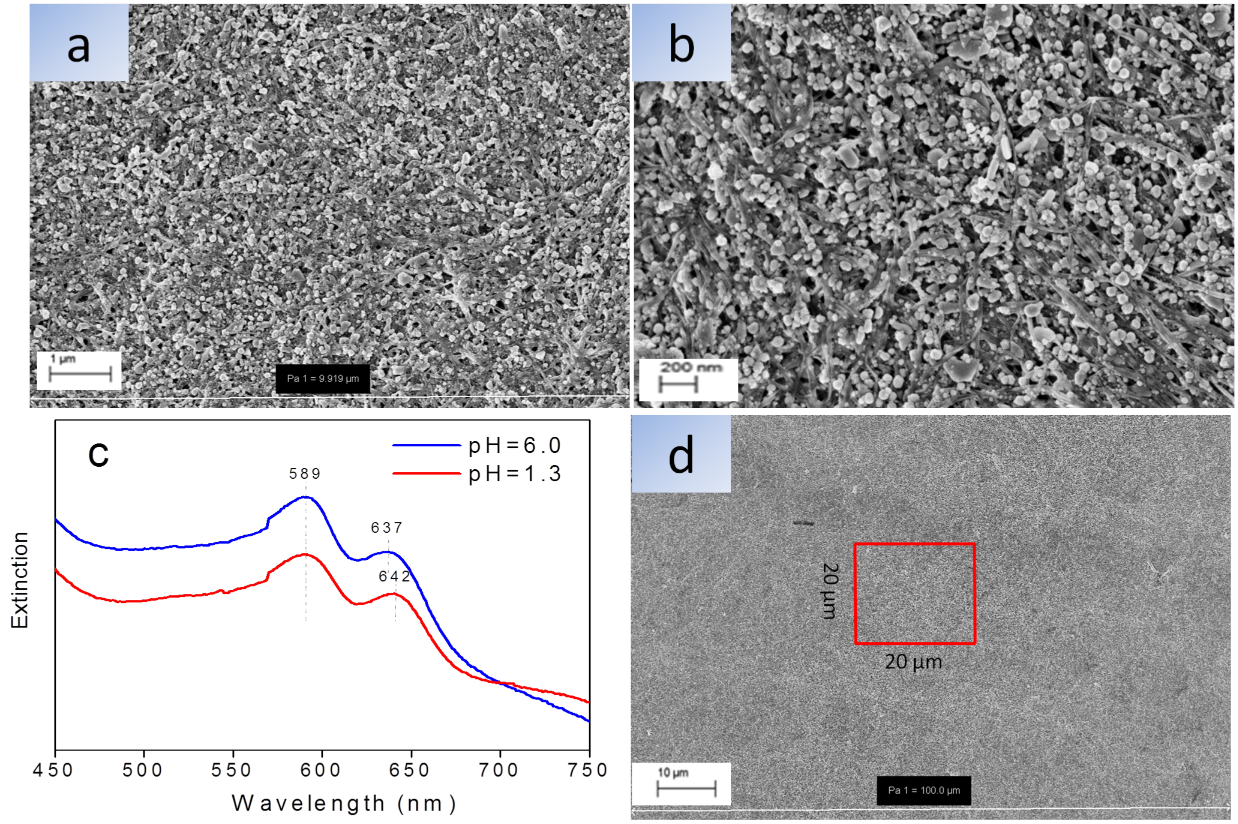

Figure S-1 SEM images of AuNP/BC dry film at a) 28000×, and b) 50000× magnification. c) Extinction spectra of AuNP/BC hydrogel exposed to aqueous solution with pH=1.3 or 6.0 for 15 s. d) SEM images of AuNP/BC dry film at 2800× magnification.

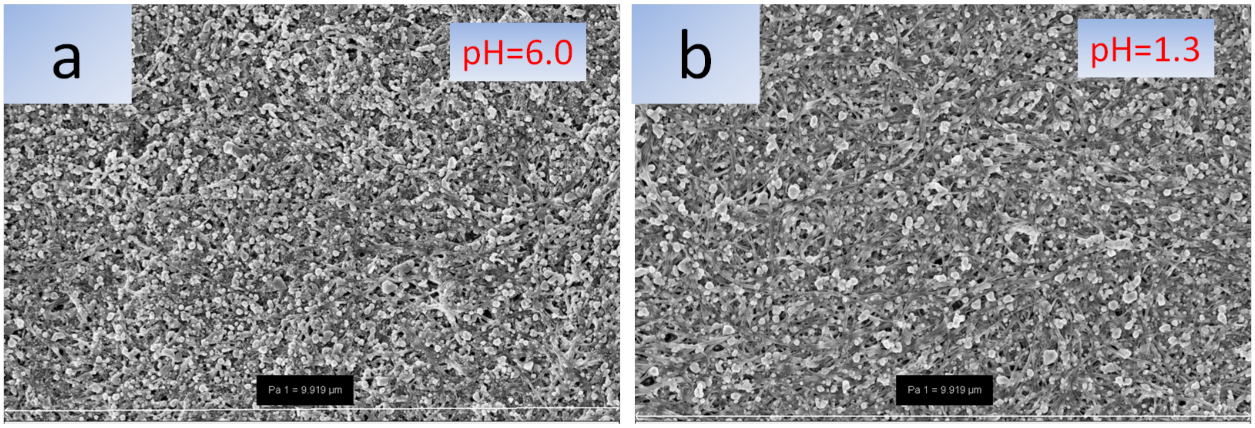

Figure S-2 SEM images of AuNP/BC dry film exposed to aqueous solution with a) pH=6.0 or b) pH=1.3 for 15 s.

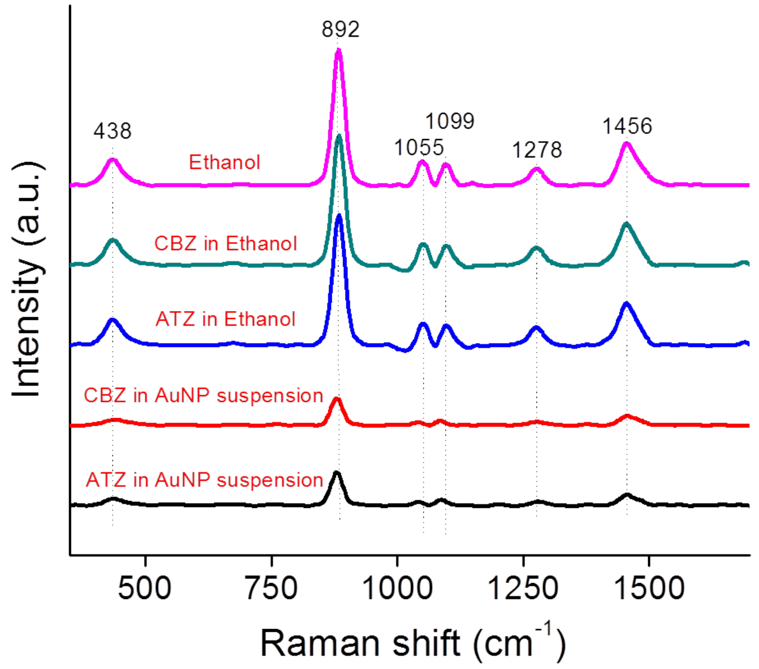

Figure S-3 Average Raman spectrum of 250 µM CBZ and ATZ in a 50 nm AuNP suspension, in an ethanol solution and pure ethanol. (Average of 400 spectra in a 100 µm × 100 µm area, laser 785 nm, 5 mW, 10× objective).

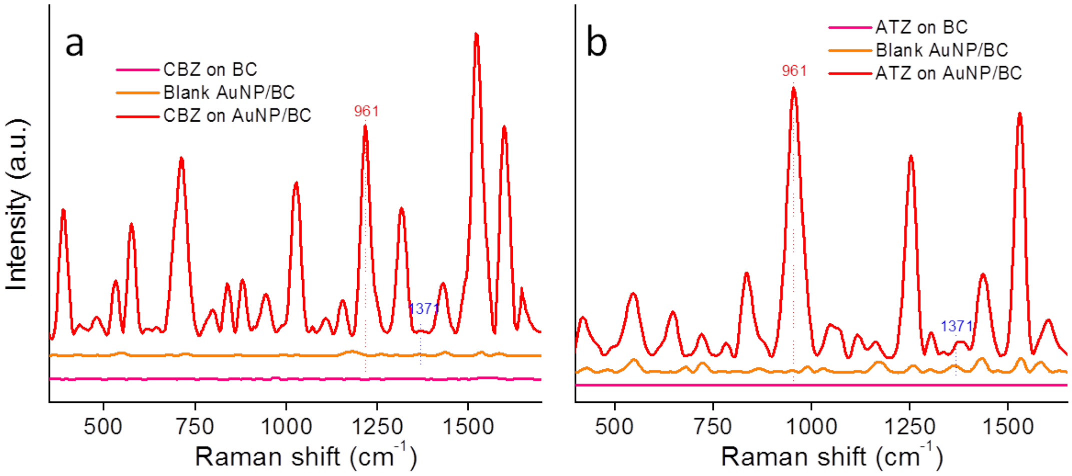

Figure S-4 Average Raman spectrum of 250 μM a) CBZ and b) ATZ on BC and AuNP/BC and blank AuNP/BC at pH=1.3 or 2.0. (Average of 400 spectra in a 20 μm × 20 μm area, laser 785 nm, 5 mW, 10× objective).

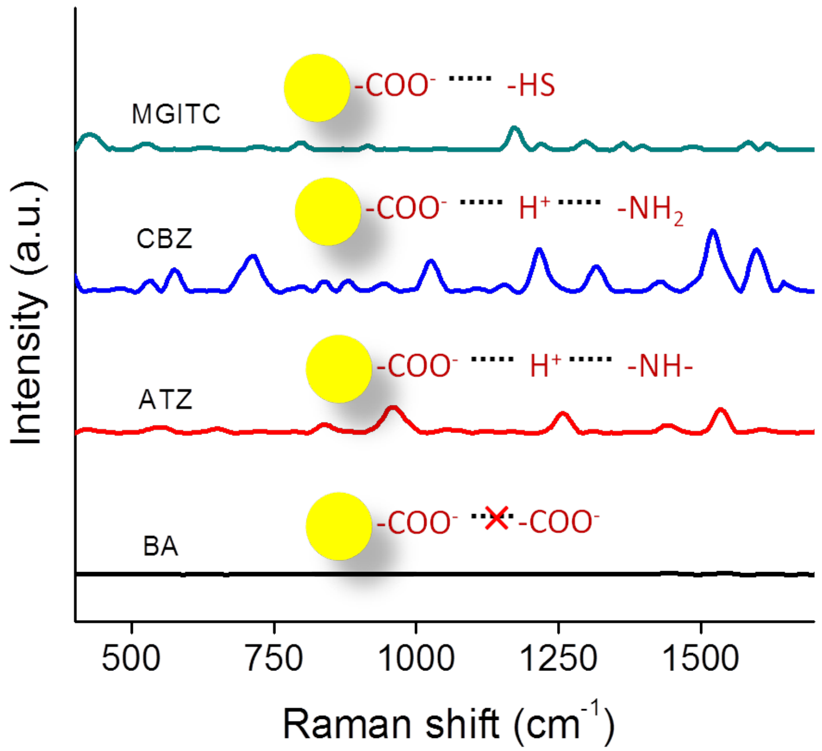

Figure S-5 Average Raman spectra of AuNP/BC exposed to 2.5 μM MGITC at pH=6.0, 250 μM CBZ and BA at pH=2.0, and 250 μM ATZ at pH=1.3.

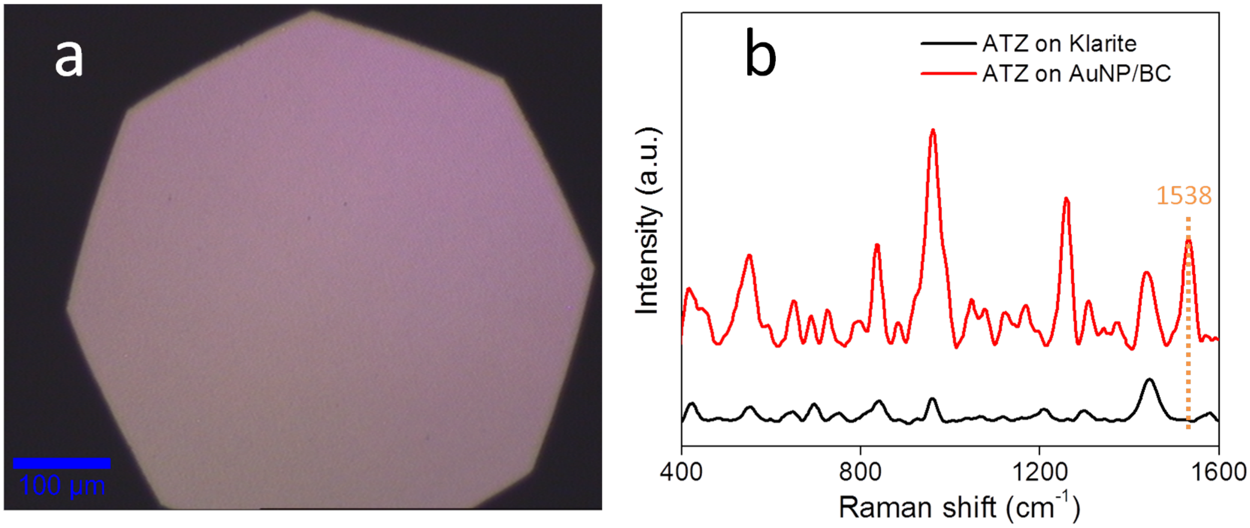

Fig. S-6 a) Optical image of Klarite; b) Average Raman spectra of atrazine (250 uM) on AuNP/BC and Klarite`s coffee ring. (Average of 400 spectra in a 100 μm × 100 μm area, laser 785 nm, 5 mW, 10× objective.)

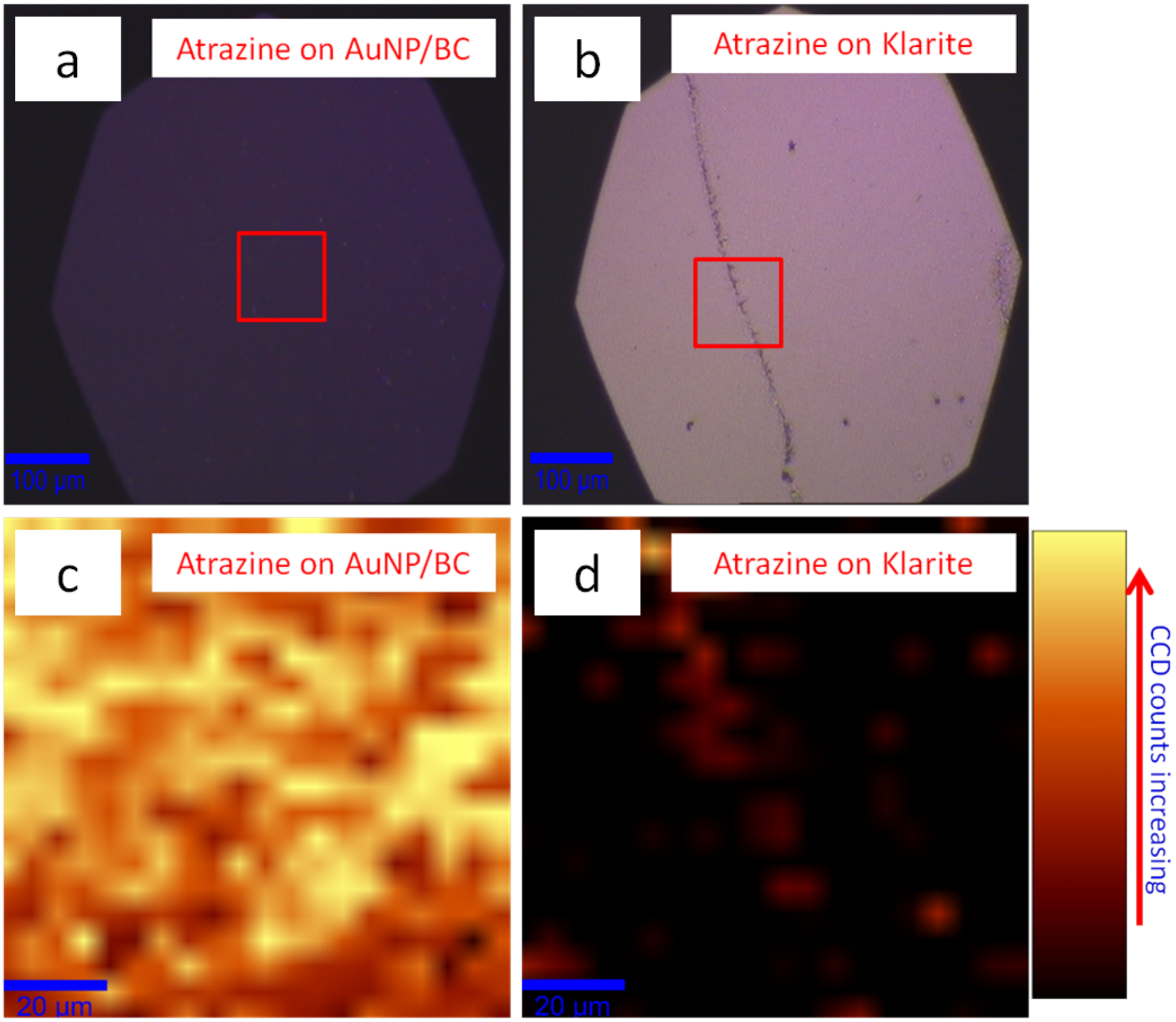

Figure S-7 Optical images of a) AuNP/BC hydrogel and b) Klarite exposed to 250 µM atrazine; Raman XY maps of the atrazine 961 cm^{-1} peak on c) AuNP/BC and d) Klarite obtained from the noted areas of the corresponding optical images. (Average of 400 spectra in a 100 µm × 100 µm area, laser 785 nm, 5 mW, 10× objective.)

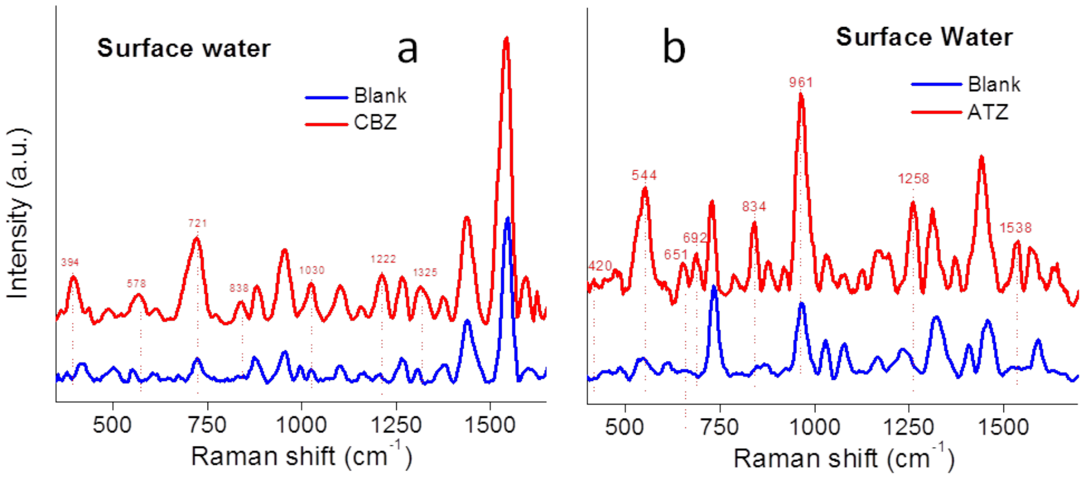

Figure S-8 Average a) CBZ and b) ATZ spectra obtained using surface water (ATZ concentration: 250 nM; CBZ concentration: 100 nM; Average of 400 spectra in a 20 μm × 20 μm area, laser 785 nm, 5 mW, 10× objective.)

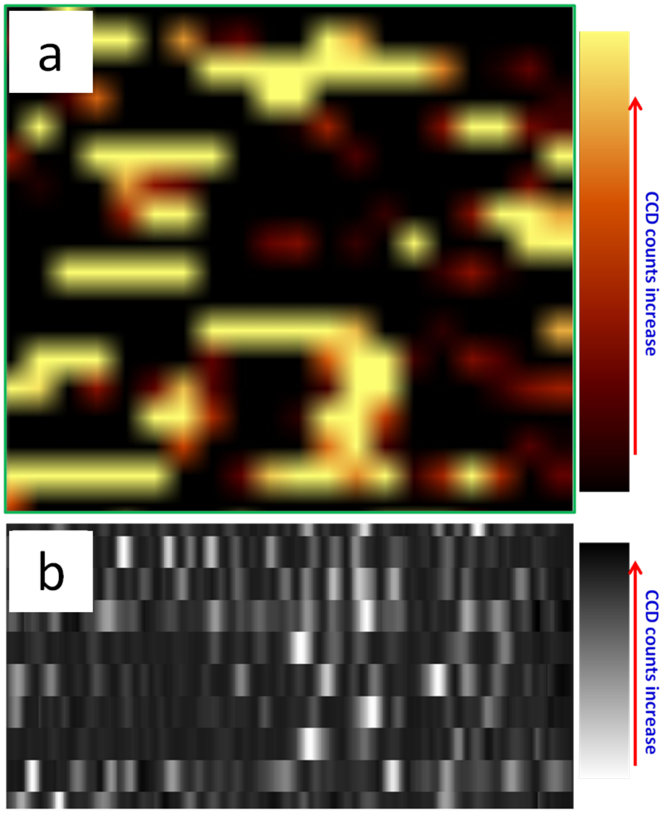

Fig. S-9 a) Raman XY map of one of the edge areas of dry AuNP/BC substrate; b) SERS barcode of 10 randomly selected spectra in the edge area. (Average of 400 spectra in a 100 µm × 100 µm area, laser 785 nm, 5 mW, 10× objective.)

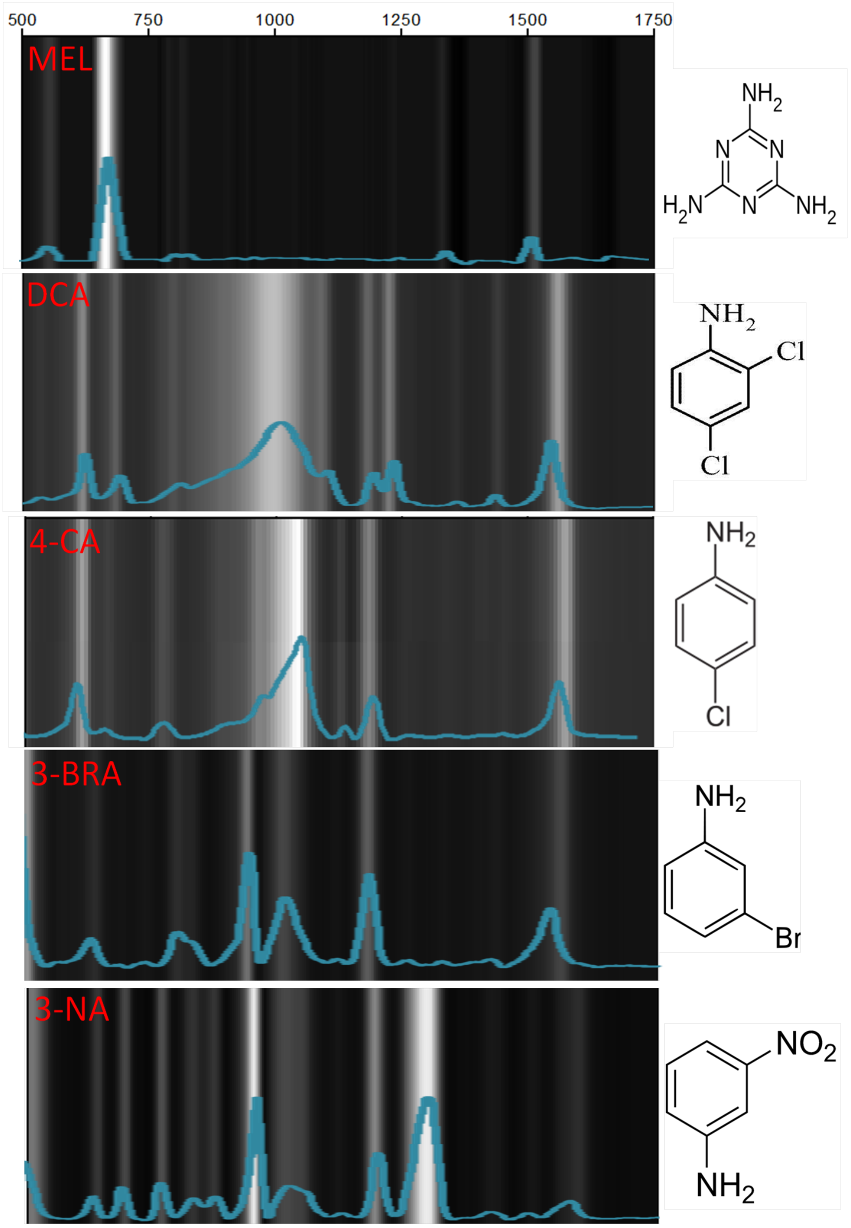

Fig. S-10 SERS barcodes of 50 randomly selected spectra in a Raman map overlapping together for melamine (MEL), 2,4-dichloroaniline (DCA), 4-chloroaniline (4-CA), 3-bromoaniline (3-BRA), and 3-nitroaniline (3-NA).

Table S-1 Assignment of the prominent peaks in normal Raman spectrum and SERS spectrum of ATZ (all assignments are based upon those defined elsewhere[1]).

Normal Raman (cm^{-1})	SERS (cm^{-1})	Assignment
647	651	τ (CH_2)
690	692	Φ (6a)
841	834	ω (CH_3) + ν(CC)
964	961	Φ (12) + ν(CC)
1257	1258	Φ (14) + τ (CH_2)
1452	1437	δ (CH_2) + δ(CH_3)
1553	1538	Φ (8a) + δ (NH)
1610	1598	δ (NH) + ν(CN)

Table S-2 Assignment of the prominent peaks in normal Raman spectrum and SERS spectrum of CBZ.

Normal Raman (cm^{-1})	SERS (cm^{-1})	Assignment
581	578	$\delta(CC)_{ring}$ [2]
723	721	$\omega(CH)$ [3]
876	883	$\nu(CCN)$ [4]
1036	1031	$\nu(C\text{-}C{=}C)$ [3]
1221	1222	$\nu(CC)$ [5]
1311	1325	$\nu(CN)$ [6]
1569	1521	δ (NH) [1]
1627	1596	δ (NH) + $\nu(CN)$ [1]

Table S-3 pH-triggered SERS detection of five additional compounds with low pK_a

Compound	pK_a	Signal peak	Background peak	$I_{signal}/I_{background}$ at pH=6.0	$I_{signal}/I_{background}$ at pH<pK_a	Enhancement*
Melamine (MEL)	5.0	703	1371	2.5	5.8	2.3×
2,4-dichloroaniline (DCA)	2.0	1584	1371	0.7	95	136×
4-chloroaniline (4-CA)	4.15	1598	1371	2	16	8×
3-bromoaniline (3-BRA)	3.58	1588	1371	0.8	6.0	7.5×
3-nitroaniline (3-NA)	2.5	1250	2143**	0.5	21	42×

*Enhancement is the ratio of $I_{signal}/I_{background}$ at pH<pK_a to that at neutral pH;
**For 3-NA, background peak is 2143 cm^{-1} because there is overlap between signal and background at 1371 cm^{-1}.

Table S-4 AuNPs embedded in various polymer matrix used for SERS detection of chemicals

Support material	Preparation Method	Application form	Application pH	Target analyte	LOD	Ref
Cellulose	Impregnation	Paper	Neutral	1,4-benzenedithiol	0.1 nM	7
Cellulose	Impregnation	Paper	Neutral	4-aminothiolphenol	1 nM	8
Cellulose	Impregnation	Paper	Neutral	1,2-bis(4-pyridyl)ethene	0.5nM	9
Cellulose /Polyvinyl chloride	Deposition + Stamping	Paper /Plastic	Neutral	4-aminothiolphenol<br>Crystal violet	1 μM<br>5 nM	10
Chitosan	Electrospinning + In situ reduction	Dry mat	Neutral	2-naphthalenethiol<br>R6G<br>Glucose	1 fM<br>1 μM<br>5 μM	11
Poly(vinyl alcohol)+ Polyethyleneimine	Electrospinning + Impregnation	Dry mat	Neutral	Rhodamine B	1 nM	12
Poly(vinyl alcohol)	Electrospinning	Dry mat	Neutral	3,3′-diethylthi-atricarbocyanine iodide	100 nM	13
Poly(acrylic acid)	Polymerization + Washing with HF	Hydrogel	Neutral	2-naphthalenethiol<br>paraquat	50nM<br>100nM	14
Poly(vinyl alcohol)	Polymerization	Dry gel	Neutral	Crystal violet	1 pM	15
Nanocellulose	In situ reduction	Dry mat	Neutral	MGITC	400 fM	16
Nanocellulose	In situ reduction	Hydrogel	Acidic	Atrazine/ Carbamazepine	11 nM/ 3 nM	**

**This study

Note 1

Comparison with commercial SERS substrate. The commercial SERS substrate Klarite was used for comparative experiments. Klarite contains ordered inverted pyramidal pits coated with gold film that exhibit a high SERS enhancement.[17] Prior to adding ATZ, the optical image for the Klarite surface was uniform (Supplementary Fig. S-6a). After a drop of ATZ (250 μM) was deposited on Klarite, a coffee ring and several black spots were observed (Supplementary Fig. S-7b), thus indicating the ATZ analyte tends to accumulate at the drop edge. Such a phenomenon is expected for many analytes with low surface affinity and supports our contention that ATZ exhibits low affinity to the AuNP surface. A XY image was centered over the coffee ring (marked in red box in Supplementary Fig. S-7b). For comparison, a randomly selected area was scanned on AuNP/BC exposed to atrazine solution at pH = 1.3 (marked in red cubic in Supplementary Fig. S-7a). The signal with the AuNP/BC substrate was uniformly distributed across the entire map, while the signal on the Klarite substrate was concentrated along the coffee ring (Supplementary Fig. S-7c&d).

Based upon the Raman intensity measurements, the Raman signal from most points on AuNP/BC is considerably stronger than the signal on the coffee ring of Klarite where the analyte concentration is very high (Supplementary Fig. S-7c&d). Such evidence indicates our AuNP/BC substrate outperforms the drop coating deposition Raman method that is known to concentrate analytes for Raman detection.[18,19] Furthermore, the nearly identical peak heights and the lack of a peak at 1538 cm^{-1} (C-N, N-H bonds; Supplementary Fig. S-6b) indicates that the ATZ molecules randomly deposit on the Klarite substrate rather than aligning in a consistent orientation on AuNP/BC through electrostatic association via their -NH- groups. Overall, the AuNP/BC can be considered a better substrate than Klarite for the following reasons: 1) it provides uniform signals without the need to look for evidence of coffee rings under the microscope; 2) it works at extremely low concentration, while Klarite only works at concentrations high enough to form visible coffee rings; 3) it is much cheaper than Klarite (estimated \$0.1 vs. > \$100) and is easier to make and optimize for large scale production.

Note 2

Synthesis of AuNPs (50 nm). AuNP seeds (13 nm) were synthesized by adding Na_3Cit into 100 mL of 1 mM boiling $HAuCl_4$ (final citrate concentration 3.88 mM) under vigorous stirring. Subsequently, 0.88 mL seed suspension and 0.44 mL of 38.8 mM Na_3Cit were added to 100 mL of boiling 0.25 mM $HAuCl_4$ with vigorous stirring. After 30 min reaction and reflux, the AuNP suspension (50 nm) was cooled to room temperature and stored at 277 K.

References

1 Costa, J. C. *et al.* Understanding the effect of adsorption geometry over substrate selectivity in the surface-enhanced Raman scattering spectra of simazine and atrazine. *J. Phys. Chem. C* **115**, 4184-4190 (2011).

2 Costa, J. C. S. *et al.* High performance gold nanorods and silver nanocubes in surface-enhanced Raman spectroscopy of pesticides. *Phys. Chem. Chem. Phys.* **11**, 7491-7498 (2009).

3 Hu, S. *et al.* Assignment of protoheme resonance Raman spectrum by heme labeling in myoglobin. *J. Am. Chem. Soc.* **118**, 12638-12646 (1996).

4 Diem, M. *et al.* Vibrational circular dichroism in amino acids and peptides. 4. vibrational analysis, assignments, and solution-phase Raman spectra of deuterated isotopomers of alanine. *J. Am. Chem. Soc.* **104**, 3329-3336 (1982).

5 Palings, I. *et al.* Assignment of fingerprint vibrations in the resonance Raman spectra of rhodopsin, isorhodopsin, and bathorhodopsin: implications for chromophore structure and environment. *Biochemistry* **26**, 2544-2556 (1987).

6 Chinsky, L. *et al.* Nucleic acid derivatives studied by preresonance and resonance Raman spectroscopy in the ultraviolet region. *Biopolymers* **17**, 1347-1359 (1978).

7 Lee, C. H. *et al.* Paper-based SERS swab for rapid trace detection on real-world surfaces. *ACS Appl. Mater. Interfaces* **2**, 3429-3435 (2010).

8 Ngo, Y. H. *et al.* Gold nanoparticle–paper as a three-dimensional surface enhanced raman scattering substrate. *Langmuir* **28**, 8782-8790 (2012).

9 Lee, C. H. *et al.* Highly sensitive surface enhanced Raman scattering substrates based on filter paper loaded with plasmonic nanostructures. *Anal. Chem.* **83**, 8953-8958 (2011).

10 Martín, A. *et al.* Flexible SERS active substrates from ordered vertical Au nanorod arrays. *RSC Adv.* **4**, 20038-20043 (2014).

11 Severyukhina, A. N. *et al.* Nanoplasmonic chitosan nanofibers as effective SERS substrate for detection of small molecules. *ACS Appl. Mater. Interfaces* **7**, 15466-15473 (2015).

12 Zhu, H. *et al.* Self-assembly of various Au nanocrystals on functionalized water-stable PVA/PEI nanofibers: a highly efficient surface-enhanced Raman scattering substrates with high density of "hot" spots. *Biosens. Bioelectron.* **54**, 91-101 (2014).

13 Zhang, C. L. *et al.* Controlled asemblies of gold nanorods in PVA nanofiber matrix as flexible free-standing SERS substrates by electrospinning. *Small* **8**, 648-653 (2012).

14 Shin, K. *et al.* Au nanoparticle-encapsulated hydrogel substrates for robust and reproducible SERS measurement. *Analyst* **138**, 932-938 (2013).

15 Yao, S. *et al.* A highly porous PVA dried gel with gold nanoparticles embedded in the network as a stable and ultrasensitive SERS substrate. *Chem. Commun. (Cambridge, U. K.)* **49**, 6409-6411 (2013).

16 Wei, H. *et al.* Preparation and evaluation of nanocellulose–gold nanoparticle nanocomposites for SERS applications. *Analyst* **140**, 5640-5649 (2015).

17 Leng, W. & Vikesland, P. J. Nanoclustered gold honeycombs for surface-enhanced Raman scattering. *Anal. Chem.* **85**, 1342-1349 (2013).

18 Halvorson, R. A. *et al.* Differentiation of microcystin, nodularin, and their component amino acids by drop-coating deposition Raman spectroscopy. *Anal. Chem.* **83**, 9273-9280 (2011).

19 Halvorson, R. A. & Vikesland, P. J. Drop coating deposition Raman (DCDR) for microcystin-LR identification and quantitation. *Environ. Sci. Technol.* **45**, 5644-5651 (2011).
